# Particulate matter hinders the development and reproduction of predatory mites of *Euseius finlandicus* (Acariformes: Phytoseiidae)

**DOI:** 10.1038/s41598-024-68570-w

**Published:** 2024-07-31

**Authors:** E. Puchalska, A. Przybysz, A. Nowak, E. Wójcik-Gront, G. Askarova, M. Lewandowski, H. Moniuszko

**Affiliations:** 1 Section of Applied Entomology, Department of Plant Protection, Institute of Horticultural Sciences, Warsaw University of Life Sciences—SGGW (WULS—SGGW), Nowoursynowska 159, 02-776 Warsaw, Poland; 2 Section of Basic Research in Horticulture, Department of Plant Protection, Institute of Horticultural Sciences, Warsaw University of Life Sciences—SGGW (WULS—SGGW), Nowoursynowska 159, 02-776 Warsaw, Poland; 3 Department of Biometry, Institute of Agriculture, Warsaw University of Life Sciences—SGGW (WULS—SGGW), Nowoursynowska 159, 02-776 Warsaw, Poland; 4 Population Ecology Lab, Faculty of Biology, Adam Mickiewicz University, Uniwersytetu Poznańskiego 6, 61‑614 Poznań, Poland

**Keywords:** Acarodomatia, Beneficial fauna, Phylloplane pollution, Phyllosphere, Phytoseiidae, Predatory mites, Zoology, Entomology, Ecology, Urban ecology

## Abstract

The foliage of the small-leaved lime (*Tilia cordata*) is characterised by the nerve axils being grown by non-glandular trichomes, which trait contributes to the enhanced retention of the particulate matter (PM). This fact may disturb the ecological service of *T. cordata* related to the structure of its leaves, which is to provide acarodomatia (micro-shelters) for the predatory mites of the Phytoseiidae family. Phytoseiids are natural enemies of a variety of plant pests, widely applied in integrated pest management (IPM). Their occurrence is largely related to acarodomatia in which these mites hide, feed, reproduce, and develop. For the first time, the influence of PM deposition within spaces typically occupied by phytoseiids is investigated. Experimental populations of *Euseius finlandicus* were reared on *T. cordata* leaves in the progressive PM-pollution. The results showed that the values of life table parameters of the predator depended significantly on the level of PM deposition on leaves. Contrary to clean leaves from the control, the medium and high contamination intensities significantly reduced the daily (by 47% and 70%, respectively) and the total fecundity (by 62% and 77%, respectively) of females which, in turn, resulted in a decreased net reproductive rate (by 67% and 81%, respectively), intrinsic rate of increase (by 40% and 55%, respectively) and finite rate of increase (by 8% and 10%, respectively) of *E. finlandicus*. The pre-ovipositional period was prolonged, while the oviposition duration was shortened and the mites matured longer. In high pollution level the mortality of phytoseiids was boosted by 19% and some females were observed with pollutant lumps adhered to the idiosoma. Also, significant shares of juvenile forms (13%) and adult females (25%) made attempts to escape from highly contaminated experimental arenas. The implications of PM retention on the shelter vegetation are discussed in the context of IPM and ecological services.

## Introduction

Despite apparent functional differences, both urban and rural ecosystems are affected by natural and anthropogenic particulate matter (PM)^1–4^. The influence which PM exerts on the local microfauna is related to its settlement on biotic (especially vegetation) and abiotic components of a given ecosystem, and it falls into two categories: (i) chemical—resulting from the content of toxic trace elements (TE-s), polyaromatic hydrocarbons (PAH-s), fertilisers and pesticides, (ii) physical—being an outcome of the presence of mineral components which mechanically affect invertebrates and their niches^5–10^. The above division systematises the ecological consequences of the urban and agricultural PM emission, such as: transportation on paved and unpaved roads, fuel combustion as well as crops and urban greenery cultivation and protection^1,4,8,11–13^. Considering that, one can assume that there are similar trends to be found in the impact of PM on organisms inhabiting the phyllosphere of municipal and rural vegetation.

The small-leaved lime (*Tilia cordata*) is a pivotal component of municipal green spaces^14,15^, it has also been recorded as a natural component of rural ecological infrastructure^16–19^, and its foliage naturally gathers a noteworthy amount of PM^20,21^. Accumulation capacity is related to the micromorphology of the lime phylloplane. The presence of trichomes, located mainly at vein junctions, and convex venation are listed among the factors contributing to enhanced PM retention^22–24^. Moreover, *T. cordata* provides microhabitats for more than a hundred species of arthropods^25,26^. Besides strictly herbivorous taxa, lime trees have been repeatedly observed as the reservoir for predatory mites of the Phytoseiidae family (Parasitiformes: Mesostigmata)^27–29^. The latter fact places limes among species eligible in the vicinity of crops, especially orchards, to which predators can freely migrate^30^. The association of the phytoseiid mites with the small-leaved lime is closely related to the presence of structures known as acarodomatia. An acarodomatium is composed of tussocks of non-glandular trichomes concentrated within pits of major leaf veins^31^. These structures are situated on the abaxial leaf side and serve as pollen and prey capture zones, ovipositional sites, and a shelter from bigger predators and unfavourable abiotic factors^31–33^.

Current data indicate that lime acarodomatia are most often utilised by such representatives of beneficial fauna as: *Amblyseius andersoni* (Chant, 1957)*, Euseius finlandicus* (Oudemans, 1915), *Typhlodromus pyri* Scheuten, 1857, *Paraseiulus soleiger* (Ribaga, 1904) and *Neoseiulella tiliarum* (Oudemans, 1930)^27,34^. Of these, *E. finlandicus* is known for its occurrence and domination on deciduous trees growing within urban environment, in forests as well as in ecological, organic and IPM (integrated pest management) orchards, and fruit plantations^27,29,35–41^. This predator is a natural enemy of spider mites, eriophyid mites, tyroglyphid mites, tarsonemid mites, small insect pests, and their eggs^42–45^. Its diet can be supplemented with pollen, nectar, fungal spores, hyphae, honeydew, and plant sap^44,46^, which qualifies *E. finlandicus* as a type IV—pollen-feeding generalist predator^47^. Unlike most phytoseiids, this species does not require the intake of proteins before successful reproduction^46^. This particular feature allows *E. finlandicus* to build up its population before pests reach outbreak levels and thus it can control the phytophages while they are still at non-damage levels^44^.

The establishment and preservation of vegetation, which provides refuge for beneficial arthropods, such as Phytoseiidae, constitutes a major pillar of IPM in cities and of sustainable agriculture in general^48–52^. Particular importance of the urban IPM results from the necessity of pesticide use limitation in densely populated municipal communities which, at the same time, greatly supports biodiversity conservation^51,53–58^. Considering that, native and/or purposely released phytoseiids deliver an ecological remedy against a variety of pests infesting ornamental plants and park trees in the cities and crops within plantations^29,35,51,54,55,59,60^.

To date, the issue of shelter plants has been raised only in the context of habitat engineering aimed at gaining optimal species arrangement and composition for the beneficial fauna to survive and reproduce^29,49–52^. Still, as already mentioned, the same trees and herbaceous plants naturally bio-filter the air from suspended solid and liquid particles^61,62^. The consequences of PM residues on the vegetation being the refuge for the natural enemies of pests have not been surveyed at all. What has been revealed so far is that different sorts of PM accumulated on cultivated and non-crop plants impact the survival rate and population growth of non-beneficial folivorous arthropods characterised by different modes of food intake and considerable resistance to pesticides^10,63–65^. Beneficial predators, in turn, such as Carabidae (Coleoptera), *Hypoaspis* spp. (Mesostigmata) and Formicidae (Hymenoptera), were investigated only with regards to soil contamination with TEs, oil derivatives, and pesticides^66–69^.

To fill the knowledge gap on the repercussions of the PM accumulation by reservoir plants settled by the predatory mites, we established a model involving species to be widely encountered within urban and horticultural environments: *E. finlandicus*—a generalist predatory mite and *T. cordata*—a phytoseiid-friendly tree providing mites with numerous foliar acarodomatia as well as showing enhanced tendency to accumulate PM. For the first time, we report on the life history parameters of *E. finlandicus* exposed to PM retained on its preferred shelter plant. We hypothesise that PM deposition on leaves, especially within hairy and tight spaces of the vein axils, makes acarodomatia impenetrable for phytoseiids, which is reflected by their reduced fitness. Therefore, PM-contaminated trees may have a limited role in providing ecosystem services as reservoirs of predatory mites.

## Results

**Figure 1:**
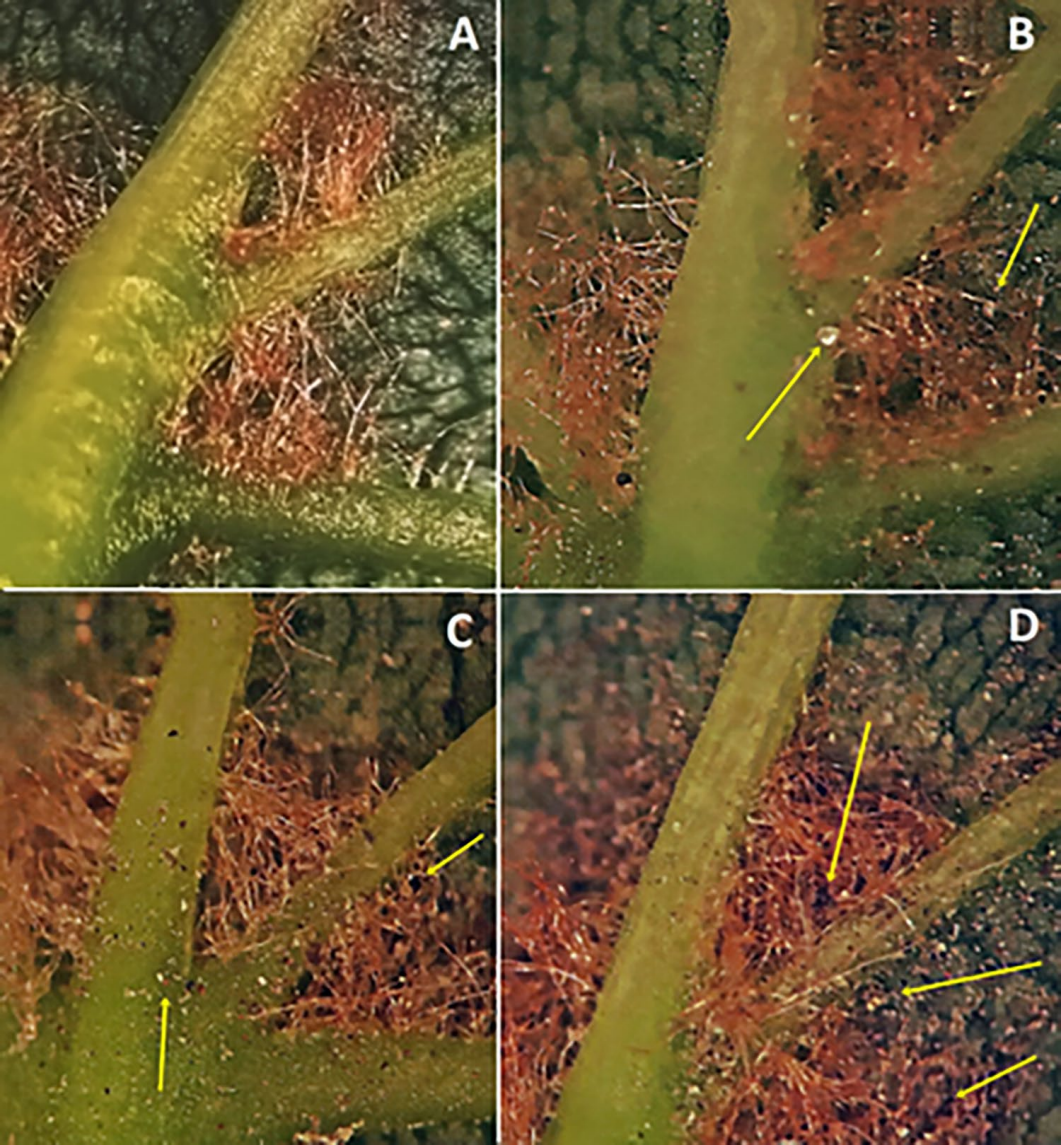
Contamination of acarodomatia with PM: (**A**)—the control; (**B**)—low level; (**C**)—medium level (**D**)—high level; arrows indicate black and whitish particles of PM.

Our observations showed that on leaves with a low level of contamination (urban park interior) there were single PM particles in the acarodomatia, which did not impede the access of phytoseiid mites. With medium leaf pollution (roadside vegetation), dust deposits were clearly visible in the acarodomatia, but these leaf structures remained, at least partially, accessible to predators. At the highest level of leaf contamination (industrial zones), most acarodomatia were blocked, remaining inaccessible to phytoseiids (Fig. 1).

*Euseius finlandicus* mites were able to complete their life cycle in all levels of pollution. The contamination intensity did not significantly influence the duration of egg and larval development (Table 1). In the case of protonymphs, deutonymphs, and the egg-to-adult duration, however, significant differences occurred, indicating a longer development time under the conditions reflecting medium (by 29%, 41%, and 20%, respectively, vs. the control) and high pollution (by 29%, 25% and 18%, respectively, vs. the control).

**Table 1:** Survival rate [%] and mean and standard deviation of developmental time [days] of immature stages of *E. finlandicus* kept in varied pollution levels; *mean values within a row followed by different letters differ significantly (*p* < 0.05).

Developmental instar	Pollution level							
	Control		Low		Medium		High	
	n	Mean ± SD	n	Mean ± SD	n	Mean ± SD	n	Mean ± SD
Egg	34	1.9 ± 0.5a*	36	2.0 ± 0.3a	34	2.0 ± 0.2a	35	2.1 ± 0.4a
Larva	33	0.9 ± 0.4a	35	1.0 ± 0.1a	32	1.0 ± 0.2a	32	1.0 ± 0.2a
Protonymph	33	1.7 ± 0.6a	34	1.9 ± 0.4a	31	2.2 ± 0.7b	31	2.2 ± 0.4b
Deutonymph	33	1.2 ± 0.5ab	34	1.1 ± 0.4a	31	1.7 ± 0.6c	30	1.5 ± 0.5bc
Egg–adult development	33	5.8 ± 0.5a	34	5.9 ± 0.5a	31	7.0 ± 0.7b	30	6.9 ± 0.6b
Egg–adult survival	35	0.94*a*	37	0.92*a*	37	0.84*a*	39	0.77*b*

The survival rate of *E. finlandicus* developing at differentiated pollution intensities is given in Table 1. In most cases, no significant differences were observed at α = 0.05. However, for the highest level of pollution, the egg–adult survival rate was significantly lower (by 17% compared to the control) than for other levels of contamination (*p* = 0.04).

Amounts of PM on the foliage significantly affected female reproduction parameters but not the sex ratio of the F1 generation (Table 2). Medium and high pollution conditions were most impactful on the mites. The females kept on control and low polluted leaves lived significantly longer (by 32% in the control vs. the highest level of pollution) and laid more eggs daily (by 70% in the control vs. the highest level of pollution) and in total (by 77% in the control vs. the highest level of pollution) (Table 2).

**Table 2:** Average and standard deviation (SD) of female longevity [days], duration of the oviposition period [days], total fecundity [eggs/female], and daily fecundity [eggs/female/day of oviposition] of *E. finlandicus* in various levels of pollution; *significantly different means (*p* < 0.05) are marked by different letters within each column.

Pollution level	n	Female longevity	Total fecundity	Daily fecundity	Preoviposition time	Oviposition time	Sex ratio F1
Control	20	21.8 ± 3.3c*	27.7 ± 6.0c	1.3 ± 0.2c	2.1 ± 0.7a	16.6 ± 3.1c	0.8 ± 0.1a
Low	18	19.1 ± 2.7bc	28.2 ± 3.2c	1.5 ± 0.2d	2.8 ± 0.5a	12.4 ± 2.3b	0.8 ± 0.1a
Medium	15	16.1 ± 2.9ab	10.6 ± 1.7b	0.7 ± 0.1b	4.0 ± 1.3b	8.3 ± 2.7a	0.7 ± 0.1a
High	15	14.9 ± 4.2a	6.5 ± 3.8a	0.4 ± 0.2a	4.5 ± 1.7b	6.0 ± 4.0a	0.7 ± 0.2a

*Euseius finlandicus* life table parameters in different conditions are given in Table 3. Significant differences occurred depending on the pollution level in all parameters except for the mean generation time (*T*). The medium and the highest contamination intensities caused a significant lowering of the net reproductive rate (*R*_0_) (by 67% and 81%, respectively, vs. the control), the intrinsic rate of increase (*r*_*m*_) (by 40% and 55%, respectively, vs. the control and the lowest pollution) and the finite rate of increase (*λ*) (by 8% and 10%, respectively, vs. the control and the lowest pollution) (Table 3).

**Table 3:** Mean and standard deviation (SD) of life table parameters (*R*_0_—the net reproductive rate, *T*—the mean length of a generation, *r*_*m*_—the intrinsic rate of increase, *λ*—the finite rate of increase) of *E. finlandicus* in different levels of pollution and multiple comparisons obtained by the bootstrap method; *significantly different means (*p* < 0.05) are marked by different letters within each row.

Parameter	Pollution level			
	Control	Low	Medium	High
*R*_0_	19.21 ± 0.71c*	19.43 ± 0.33*c*	6.35 ± 0.28*b*	3.80 ± 0.50*a*
*T*	15.07 ± 0.31*a*	14.89 ± 0.19*a*	14.80 ± 0.37*a*	14.52 ± 0.38*a*
*r*_*m*_	0.20 ± 0.01*c*	0.20 ± 0.01*c*	0.12 ± 0.01*b*	0.09 ± 0.01*a*
*λ*	1.22 ± 0.01*c*	1.22 ± 0.01*c*	1.13 ± 0.01*b*	1.10 ± 0.01*a*

From an initial absence of escapees in the control group, there was a statistically significant increase to 13% for immature individuals of *E. finlandicus* and 25% for females in medium and high levels of pollution (Table 4).

**Table 4:** Shares of the immature and adult mites which escaped experimental arenas; *significantly different shares are marked by different letters within each column.

Pollution level	Escape rate of immatures	Escape rate of females
Control	0%a*	0%*a*
Low	8%*ab*	10%*ab*
Medium	8%*ab*	25%*b*
High	13%*b*	25%*b*

**Figure 2:**
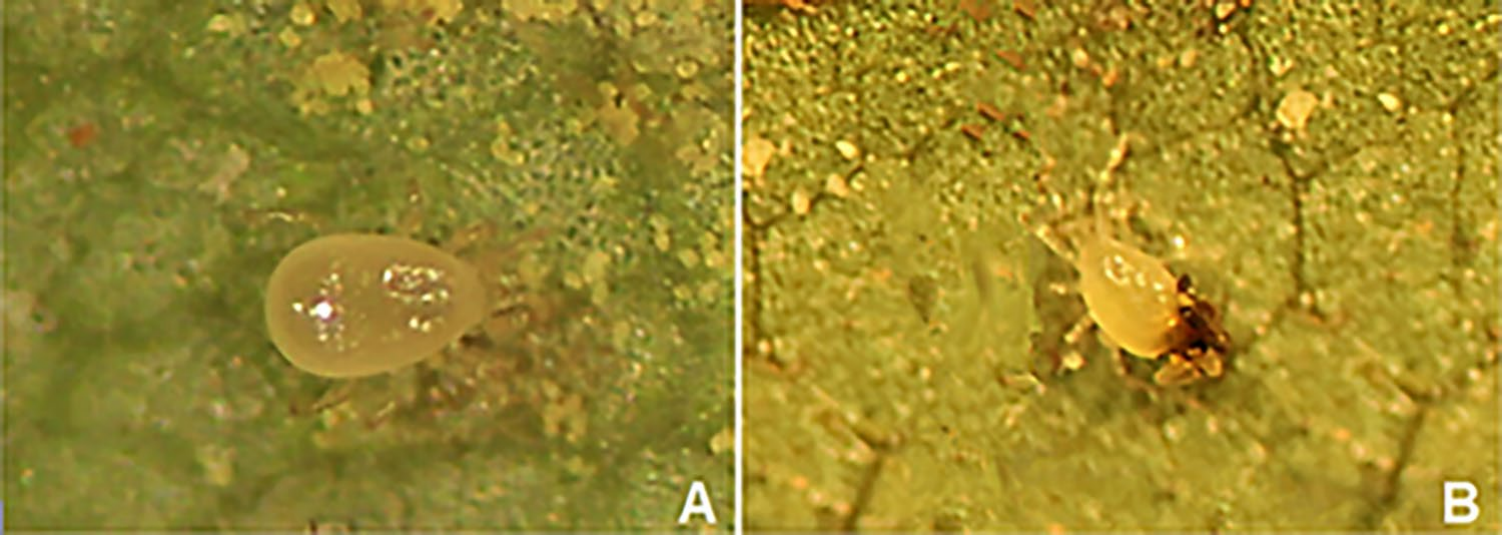
*Euseius finlandicus* females: (**A**)—PM-free feeding on pollen (control); (**B**)—with pollution articles adhered to idiosoma (the highest level of pollution).

Contrary to the control females and those from low and medium levels, the females kept in high pollution level were repeatedly observed to be covered by pollution particles, despite their introduction to the leaves only following the application of PM (Fig. 2).

## Discussion

The results confirm the assumption that leaf structures that constitute acarodomatia work against predatory mites in the presence of PM as the pollutant tends to accumulate within hairy vein branches^23,24^. At the same time, unavailability of acarodomatia has a detrimental impact on the developmental and reproductive parameters of *E. finlandicus*. The females from the medium and high contamination levels were characterised by a significantly longer period preceding oviposition. Egg deposition was delayed, probably due to the attempts of females to find another, available and clean, ovipositional site, which was impossible due to the presence of PM particles. Devoting more effort to searching for suitable egg-laying sites may have increased the total movement time of females and reduced their fecundity. When the females finally started to lay eggs in the polluted domatia, their oviposition time was significantly shorter than on control leaves. In consequence, those females had considerably lower daily (by 47% and 70%, respectively) and total (by 62% and 77%, respectively) fecundity. Finally, prolonged egg-to-adult development can also be attributed to the inaccessibility of foliar domatia, as these structures are utilised not only by the gravid females^33,70,71^ but also by the juvenile Phytoseiidae^72^. Immature individuals need shelters wherein they can moult undisturbed to the next developmental stage^73^.

Laboratory experiments indicated that the total blockage of foliar domatia by artificially applied bitumen paint reduced the number of phytoseiids by 76% per leaf of the blueberry ash, *Elaeocarpus reticulatus* (Oxalidales: Elaeocarpaceae)^70^. Similarly, application of pruning tar on acarodomatia located within the frost grape, *Vitis riparia* (Vitales: Vitaceae) foliage resulted in the gravid females of *T. pyri* and *A. andersoni* depositing fewer eggs and relocating for the leaf zones with undisturbed shelters^71^. It agrees with our observations on the fecundity but also on the behaviour of females. In the control combinations all the females introduced to the arenas remained there for the rest of their lives, while in the lowest pollution 10% of escapes were recorded—the mites stuck within the adhesive barrier. In the medium and high pollution, in turn, as many as 25% of the females attempted to leave the experimental arena, which constituted a statistically significant share. Moreover, in the case of control and low-contaminated foliage also the immature individuals were observed to spend more time in acarodomatia, compared to relatively active juveniles from the medium- and highly-polluted leaves. These mites often scanned the arena's surface and repeatedly approached the adhesive barrier, perhaps looking for an escape route. This resulted in a significant percentage (13%) of the juveniles attempting to desert the highly polluted experimental arenas. Mites’ attempts to withdraw from the PM-contaminated environment may have been caused by two reasons: (i) the above-discussed blockage of acarodomatia resulting in the shortage of ovipositional and moulting sites, (ii) there are premises that TEs and organic compounds (solid products of liquid fuels combustion) contained in PM possess repellent properties against Phytoseiidae. Soudy et al.^74^ researched the impact of progressing concentrations (12.5, 25 and 50 ppm) of Cd, Cu, Zn and As on IPM predators: *Phytoseiulus persimilis* Athias-Henriot, 1957 and *Amblyseius swirskii* Athias-Henriot, 1962. The authors revealed that tested elements (also present in the road dust used in our research) provoked the two phytoseiid species to leave the polluted sites (Cd repellent rate: 12–69% and 13–33%; Zn: 7–9% and 2–13%; Cu: 11–22% and 9–19%; As: 10–14% and 12–28% in *P. persimilis* and *A. swirskii*, respectively).

*Euseius finlandicus* is also known to replenish fluids by puncturing the leaf blade and sucking the cell sap^47^. This may have resulted in the ingestion of toxic TEs (As, Cd, Cu, Ni, Pb and Zn), which were initially incorporated by the leaf tissues, as it was discussed in the case of *Tetranychus urticae* Koch, 1836 (Acariformes: Prostigmata)^10^. Of the listed elements, As, Cd, Cu and Zn were confirmed to increase the mortality of *A. swirskii* and *P. persimilis*^74^. Moreover, in our experiments, the mites were provided with pollen deposited on the leaf surface as a supplementary food source. We speculate that the fat-containing pollen grains absorb the lipophilic PAHs^75^, which might have contributed to the mites poisoning. The synergistic effect of both types of toxins could have resulted in significantly higher mortality of the mites kept on the most heavily contaminated foliage.

The observed adherence of PM lumps to the bodies of *E. finlandicus* mites (Fig. 2B) could be a result of their attempts to penetrate PM-clogged domatia. Body pollution was not observed in *T. urticae* reared on PM-contaminated *T. cordata* foliage^10^. Moreover, the latter species developed a strategy aimed at PM avoidance which involved suspending the pollution lumps on the silken threads produced by the spider mites^10^. This observation indicates that not only does PM slow down the population increase of the beneficial mite, but it also turns out that one of the main agricultural pest performs better in the presence of toxins and mechanical hindrances. This is also reflected by the difference in the scale of fecundity decrease between the two species^10^. Exposure of the spider mites to the medium and high amounts of PM resulted in their daily and total fecundity being reduced to a lesser extent compared to *E. finlandicus* (daily fecundity: 22% vs. 47% (medium) and 26% vs. 70% (high); total fecundity: 32% vs. 62% (medium) and 30% vs. 77% (high)). Also the net reproductive rate, the intrinsic rate of increase and the finite rate of increase of *E. finlandicus* were reduced by the PM several times more than those of the spider mite. Finally, in contrast to the studied phytoseiid species, PM did not impact the mortality rate and development time of *T. urticae*^10^.

The above-cited study on the spider mite and PM pollution was concluded by the prediction that PM would have much stronger influence on other, less resilient than *T. urticae*, invertebrates, which has proven true. The discrepancy in the harmfulness of PM to beneficial and pest species is so great that it may imply the necessity of reapproaching some aspects of the agricultural and urban IPM. The integrated pest management, the biological component of which involves the introduction and/or support of natural enemies aimed at pesticide use reduction, relies, among others, on the urban and rural green infrastructure and sheltering services it provides^49–52,57^. However, research on reservoir plants seems to be neglected in the context of the inevitable PM retention. Our results indicate that it is a considerable omission.

The accumulated PM obstructs the vegetation in providing ecosystem services of the beneficial mites sheltering. The relevancy of this phenomenon results from the fact that Phytoseiidae comprise a fundamental group within IPM arthropods^37,42,45,54^. Despite the difference in the application scale between urban and agricultural IPM^57^, the necessity to increase pest bio-control, thus limiting the pesticide use, remains current^58^. In addition, some researchers maintain that there are insufficient interdisciplinary inquiries on the ecological aspects of IPM^76^. The present research contributes to reduction of this knowledge gap by providing a model of PM pollution mechanisms which can be adapted to urban and rural environments. The results suggest that urban greenery as well as ecological green areas in agrocoenoses, may provide ecosystem services and act as a reservoir of natural enemies when the degree of PM contamination of leaves is low, i.e., comparable to or lower than that characteristic of the city park interior. In this context, the creation of clean transport zones in cities takes on new significance. In more polluted areas, in turn, shelter plants can play their role by growing inside dense groups of vegetation (parks in cities, mid-field clusters of trees or forests in agrocenoses), so that the outer rows of trees play the role of a screen stopping PM. Such an approach is supported by the data on unequal PM distribution on the roadside vegetation which decreases at increasing distance from the road^77–79^.

The negative impact of PM accumulating on shelter plants on *E. finlandicus* suggests that this is a factor that may also affect other species of predatory mites. It is possible that diverse Phytoseiidae species may respond differently to PM contamination, therefore there is a need for further research to assess the sensitivity of individual species to this type of pollution. The results indicate that this may be an important factor determining the species composition of indigenous phytoseiid mites and thus the efficiency of conservation biological control.

### Study limitation

The authors are aware that a limitation of this study is the inability to clearly distinguish whether the disturbances in the development and reproduction of phytoseiid mites resulted from PM toxicity or from physical obstruction of acarodomatia caused by PM. However, our intention was to create conditions reflecting real life in which a number of factors are typically involved. In our view, both toxicity and mechanical obstruction matter and their impacts should be considered synergistic. Heterogeneous nature of PM, its abundance and tendency to accumulate on shelter vegetation makes this pollutant a complex factor affecting the development and functioning of beneficial fauna in more than one way.

## Material and methods

### Experimental model

The plant material was *Tilia cordata* (Malvales: Malvaceae) the foliage of which provides acarodomatia frequently utilised by *E. finlandicus* mites. Leaves, similar in age and size, originated from apparently uncontaminated lime trees growing in the centre of the Warsaw University of Life Sciences campus (Warsaw, central Poland). Following collection plant material was selected to exclude damaged and infected leaves, and additionally gently rinsed under running water to remove potential pollution as well as pests and beneficial arthropods. Subsequently, the foliage underwent artificial contamination with PM (road dust containing As, Ba, Cd, Cr, Cu, Fe, Mn, Ni, Pb, Pt and Zn; screened with X-ray fluorescence spectrometry analyzer Vanta Vac 3C Series (Olympus, Tokyo, Japan). Pollutant was applied according to the protocol described in Moniuszko et al.^10^, i.e., predetermined PM densities (given below) were achieved by evenly applying specific quantities of dust on specific areas on which leaves were placed. As a result, leaves were covered by PM with progressive intensity:

control: rinsed leaves only,

(I) low: 20 µg cm^−2^ of PM; contamination intensity corresponding to the urban park interior,
(II) medium: 40 µg cm^−2^ of PM; contamination typical for roadside vegetation,
(III) high: 85 µg cm^−2^ of PM; pollution intensity to be found within heavily contaminated industrial zones.

*Euseius finlandicus* (Parasitiformes: Mesostigmata) mites were initially collected from *T. cordata* trees growing in Kabacki Forest (Warsaw, Poland). Before the experiment, a mass culture of the mites was established and ran through five–six generations. *Euseius finlandicus* individuals were kept in rearing containers (25 cm × 21 cm × 7 cm) filled with a wet sponge (19.5 cm × 18.5 cm × 3.5 cm) topped with a plastic plate. Edges of the plate were covered with 1.5 cm wide filter paper onto which a layer of fruit tree grease (Vitax®) was applied. In such a configuration the mites were provided with access to water and, simultaneously, were prevented from escaping. In the centre of each plate, several oviposition units (tent-shaped pieces of transparent plastic filled with cotton threads) were placed. Mites were fed with the holm oak pollen (*Quercus ilex*) obtained from CCREA—DC (Council for Agricultural Research and Economics—Research Centre for Plant Protection and Certification, Italy) and collected according to the protocol described in Sabbatini Peverieri et al.^80^. Cultures of *E. finlandicus* were kept in a climate chamber (Sanyo MLR-350H, Japan) at 25 °C temperature, relative humidity of 70 ± 10%, and 16/8 (day/night) photoperiod, in the Section of Applied Entomology laboratory (Department of Plant Protection, Institute of Horticultural Sciences, Warsaw University of Life Sciences).

### Development and reproduction of *E. finlandicus* versus PM contamination

Clean (the control) and PM-polluted (experimental groups with I–III levels of contamination) small-leaved lime leaves were placed upside down on wet cotton wool which tightly filled 9 cm Petri dishes (one leaf per dish). Moist material, watered daily, prevented the foliage from losing turgor. Experimental arenas within each leaf were restricted with a layer of Vitax® glue (3 cm diameter). The leaf base and three first-degree vein branches, which constituted the largest acarodomatia, were included in each arena. Fresh portions of the holm oak pollen were provided as a food source and restocked every third day. A dose of approximately 0.56 mg of pollen was replaced in the same spot in the arena, away from the acarodomatia.

To assess the duration and mortality rate of each developmental instar of *E. finlandicus* about the level of contamination, single gravid females, previously synchronised in terms of age, (n = 40 per pollution level and the control; 160 individuals in total) were left for 24 h in experimental arenas for oviposition. A single egg was left in each arena from the eggs deposited, while the females and other eggs were removed. Hatched mites were being observed daily until death or emergence of the adult instars. The presence of an exuvium was interpreted as a confirmation of successful moulting to the next developmental stage.

Data on female lifespan, daily and total fecundity as well as oviposition period were obtained using analogical to the above-described experimental arenas (n = 20 per combination; 80 individuals in total). In every arena, one newly-emerged female was placed and coupled with a male from a stock culture. In the case of a male's death, a new one was added. The number of eggs laid by an individual female was recorded daily until the female died. Every day counted eggs were removed from the arenas and placed in a new unit to record the sex ratio of the progeny.

All life table experiments were carried out in the environmental chamber (25 °C, RH 70 ± 10%, 16:8 day/night).When determining the average values of individual developmental and reproductive parameters, trials in which individuals died as a result of drowning in the glue marking the experimental arena were not taken into account. Such events (observed only in PM-contaminated trials) were treated as escape attempts.

### Statistical evaluation

The statistical analysis of the results involved the utilisation of the STATISTICA version 13.0 software^81^. To assess the normality of the sample distribution, the Kolmogorov–Smirnov test was employed. Since the data did not conform to a normal distribution, the Kruskal–Wallis non-parametric ANOVA test was selected for evaluating the variations in estimated variables in *E. finlandicus* between pollution levels. After the Kruskal–Wallis test, Dunn's post-hoc test was applied to discern the groups that exhibited significant differences among them. The Z-score test was used for sex ratio of F1 generation, egg-adult survival, and the shares of the immature and adult mites that escaped experimental arenas. Throughout the analysis, statistical significance was considered to be attained if the p-value derived from these tests was less than 0.05.

Life tables of *E. finlandicus* kept in differently contaminated environments were constructed based on the observed age-specific survival rate (percent of surviving females at the instant *x*) and age-specific fecundity rate (number of eggs laid by a female per day). The method established by Birch (1948)^82^ was used to calculate the net reproductive rate (*R*_0_), the mean length of a generation (*T*), the intrinsic rate of increase (*r*_*m*_), and the finite rate of increase (*λ*). To estimate standard deviations and perform multiple comparisons of the pollution levels based on life table parameters the bootstrap method was utilised^83^.

## Data Availability

The datasets generated during and/or analysed during the current study are available from the corresponding authors on reasonable request.
